# Measuring and addressing violence against women with disabilities in Africa

**DOI:** 10.4102/ajod.v14i0.1576

**Published:** 2025-02-24

**Authors:** Clifford Odimegwu, Obasanjo A. Bolarinwa, Yusuff Adebisi

**Affiliations:** 1 Department of Demography and Population Studies, Faculty of Social Sciences, University of the Witwatersrand, Johannesburg, South Africa; 2 Department of Public Health, York St John University, London, United Kingdom; 3 Department of Education, College of Social Science, University of Glasgow, Glasgow, United Kingdom

**Keywords:** violence, women with disabilities, Africa, data collection, sexual health rights

## Abstract

Violence against women with disabilities (VAWDs) in Africa is a significant issue, with women facing higher risks of domestic, sexual and gender-based violence. However, data on VAWD are scarce, hindering effective policy development. Challenges include the lack of disaggregated data by sex and disability, methodological gaps and the absence of culturally relevant measurement tools. Common tools such as the Washington Group Short Set on Functioning often miss important nuances. To improve data accuracy, integrating comprehensive disability measures into national surveys and developing context-specific tools are essential. Accurate data are crucial for creating policies to reduce violence and protect women with disabilities in Africa.

## Introduction

The issue of violence against women with disabilities (VAWDs) in Africa demands urgent attention. Globally, over 600 million people live with disabilities, with 80 million residing in Africa (African Studies Centre Leiden 2024). Among these, women and girls with disabilities face a significantly higher risk of violence. Studies both in Africa (Chirwa et al. 2020) and globally (Dunkle et al. 2018) reveal that women and girls are twice as likely to experience domestic violence and other forms of sexual and gender-based violence when you compare to those without disabilities. Discrimination, stigma and restrictive social norms amplify the vulnerability of women with disabilities to violence and complicate efforts to collect accurate data. A recent World Health Organization (WHO) report (WHO 2024) stresses the importance of examining these issues to improve the measurement and understanding of VAWD in Africa.

Data on VAWD in Africa are scarce and often lack the necessary detail to inform effective policies and interventions compared to high-income countries, such as the United States and the United Kingdom, which have institutionalised systems for collecting detailed, disaggregated data on violence against women, including those with disabilities. For example, national surveys such as the National Crime Victimization Survey (NCVS) and the Crime Survey for England and Wales (CSEW) routinely collect comprehensive data, providing insights into the prevalence, types and circumstances of violence (Bureau of Justice Statistics 2020; Office for National Statistics 2021). In contrast, many African datasets and national surveys do not disaggregate data by sex and disability, leading to significant underestimation of the prevalence and impact of violence against women with disabilities. To improve data availability, it is essential to integrate comprehensive disability measures into national surveys and ensure that data collection processes are inclusive and accessible. Similarly, several methodological issues hinder effective VAWD measurement. An additional problem is the lack of standardised definitions and tools for measuring both violence and disability. Many studies do not specify the forms of violence assessed, and when they do, they often omit disability-specific types, such as denial of care or withholding assistive devices. Tools such as the Washington Group Short Set on Functioning (WHO 2024), while widely used, have limitations in sensitivity and scope, often missing mild to moderate disabilities and participation domains (Bolarinwa, Odimegwu & Adebisi 2024).

Another significant challenge is the lack of measurement tools tailored to Africa’s unique cultural, social and economic contexts. This absence results in data collection that may lack accuracy and relevance, ultimately hindering effective interventions and policies. To obtain accurate data on women’s experiences in Africa, it is imperative to address methodological shortcomings by adopting good practices. Data collection instruments should be chosen based on research purpose, feasibility and specific needs. Tools such as the WHO Disability Assessment Schedule and the Functioning and Disability Disaggregation Tool could offer more details (WHO 2024). Developing context-specific measurement tools for Africa is crucial. Surveys should address barriers women with disabilities face in reporting violence and include diverse perspectives. Adequate training for survey teams is essential to ensure ethical, safe and accessible data collection. This approach ensures women can participate without facing additional risks (Sabariego et al. 2015).

To contextualise the application of ideas expressed in this article, we underscore some recent examples. For instance, integrating comprehensive disability measures into national surveys, as demonstrated in the 2022 census conducted in South Africa, which included expanded disability questions, demonstrates how comprehensive disability data can inform targeted interventions against violence (DataFirst 2022; WHO 2024). Contextualising measurement tools to reflect Africa’s unique socio-cultural realities is essential, and locally adapted methodologies, such as community-based participatory approaches, have shown promise in improving the accuracy and relevance of data collection while empowering marginalised groups (Chirwa et al. 2020).

The challenges of reporting violence, often compounded by stigma and societal norms, require culturally sensitive strategies. However, engaging trusted community leaders and leveraging informal networks have been identified as effective mechanisms to enhance the participation of women with disabilities in violence surveys (Bolarinwa et al. 2025; Dunkle et al. 2018). Furthermore, strengthening legislative frameworks supported by comprehensive disability-disaggregated data enhances the implementation and enforcement of laws designed to protect these women (WHO 2024).

## Conclusion

Improving VAWD measurement in Africa requires addressing data availability, methodological issues and implementing best practices. Inclusive, accessible data collection, paired with ethical considerations, is key to obtaining accurate data. This information is crucial for developing interventions and policies to protect women with disabilities, significantly reducing violence and advancing gender equality. By embedding these context-specific practices, this opinion article offers a roadmap for reducing violence against women with disabilities and advancing gender equality across the continent.

## References

[CIT0001] African Studies Centre Leiden, 2024, *Disability in Africa*, viewed 21 June 2024, from https://www.ascleiden.nl/content/webdossiers/disability-africa.

[CIT0002] Bolarinwa, O.A., Odimegwu, C. & Adebisi, Y.A., 2024, ‘Leaving no one behind: Addressing the sexuality of people with disabilities’, *International Journal for Equity in Health* 23(1), 129. 10.1186/s12939-024-02219-y 38937839 PMC11212267

[CIT0003] Bolarinwa, O.A., Odimegwu, C., Babalola, B.I. & Mohammed, A., 2025, ‘A qualitative study exploring the sexual experiences of women with disabilities in Lagos, Nigeria’, *Sexuality and Disability* 43(1), 1–25. 10.1007/s11195-024-09881-8

[CIT0004] Bureau of Justice Statistics, 2020, *National Crime Victimization Survey (NCVS)*, viewed 16 January 2025, from https://bjs.ojp.gov.

[CIT0005] Chirwa, E., Jewkes, R., Van Der Heijden, I. & Dunkle, K., 2020, ‘Intimate partner violence among women with and without disabilities: A pooled analysis of baseline data from seven violence-prevention programmes’, *BMJ Global Health* 5(11), e002156. 10.1136/bmjgh-2019-002156 PMC7677328 33208311

[CIT0006] DataFirst, 2022, *South African Census 2022, 10% Sample*, University of Cape Town, viewed 16 January 2025, from https://www.datafirst.uct.ac.za/dataportal/index.php/catalog/982.

[CIT0007] Dunkle, K., Van Der Heijden, I., Stern, E. & Chirwa, E., 2018, *Disability and violence against women and girls: What works to prevent violence against women and girls global programme*, pp. 1–6, Department for International Development, London.

[CIT0008] Office for National Statistics (ONS), 2021, *Crime survey for England and Wales: Domestic abuse, disability, and vulnerability*, viewed 16 January 2025, from https://www.ons.gov.uk.

[CIT0009] Sabariego, C., Oberhauser, C., Posarac, A., Bickenbach, J., Kostanjsek, N., Chatterji, S. et al., 2015, ‘Measuring disability: Comparing the impact of two data collection approaches on disability rates’, *International Journal of Environmental Research and Public Health* 12(9), 10329–10351. 10.3390/ijerph120910329 26308039 PMC4586614

[CIT0010] World Health Organization (WHO), 2024, *Measuring violence against women with disability*, viewed 21 June 2024, from https://www.who.int/publications/i/item/9789240089563.

